# HHCDB: a database of human heterochromatin regions

**DOI:** 10.1093/nar/gkad954

**Published:** 2023-10-28

**Authors:** Hongli Wang, Mu Su, Jie Xing, Jie Zhou, Jinzhang Wang, Long Chen, Haomin Dong, Wenhui Xue, Yubo Liu, Qiong Wu, Yan Zhang

**Affiliations:** School of Life Science and Technology, Harbin Institute of Technology, Harbin 150001, China; School of Life Science and Technology, Harbin Institute of Technology, Harbin 150001, China; School of Life Science and Technology, Harbin Institute of Technology, Harbin 150001, China; School of Life Science and Technology, Harbin Institute of Technology, Harbin 150001, China; School of Life Science and Technology, Harbin Institute of Technology, Harbin 150001, China; School of Life Science and Technology, Harbin Institute of Technology, Harbin 150001, China; School of Life Science and Technology, Harbin Institute of Technology, Harbin 150001, China; School of Life Science and Technology, Harbin Institute of Technology, Harbin 150001, China; The Leicester International Institute, Dalian University of Technology, Dalian 116000, China; School of Life Science and Technology, Harbin Institute of Technology, Harbin 150001, China; School of Life Science and Technology, Harbin Institute of Technology, Harbin 150001, China; College of Pathology, Qiqihar Medical University, Qiqihar 161042, China

## Abstract

Heterochromatin plays essential roles in eukaryotic genomes, such as regulating genes, maintaining genome integrity and silencing repetitive DNA elements. Identifying genome-wide heterochromatin regions is crucial for studying transcriptional regulation. We propose the Human Heterochromatin Chromatin Database (HHCDB) for archiving heterochromatin regions defined by specific or combined histone modifications (H3K27me3, H3K9me2, H3K9me3) according to a unified pipeline. 42 839 743 heterochromatin regions were identified from 578 samples derived from 241 cell-types/cell lines and 92 tissue types. Genomic information is provided in HHCDB, including chromatin location, gene structure, transcripts, distance from transcription start site, neighboring genes, CpG islands, transposable elements, 3D genomic structure and functional annotations. Furthermore, transcriptome data from 73 single cells were analyzed and integrated to explore cell type-specific heterochromatin-related genes. HHCDB affords rich visualization through the UCSC Genome Browser and our self-developed tools. We have also developed a specialized online analysis platform to mine differential heterochromatin regions in cancers. We performed several analyses to explore the function of cancer-specific heterochromatin-related genes, including clinical feature analysis, immune cell infiltration analysis and the construction of drug-target networks. HHCDB is a valuable resource for studying epigenetic regulation, 3D genomics and heterochromatin regulation in development and disease. HHCDB is freely accessible at http://hhcdb.edbc.org/.

## Graphical Abstract

**Graphical Abstract:**
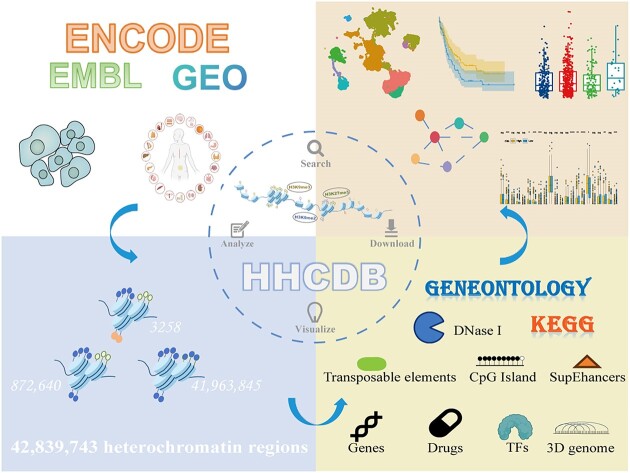

## Introduction

Heterochromatin is a crucial feature of eukaryotic genomes, participating in the maintenance of genome integrity, regulation of gene expression, cell fate determination and silencing of repetitive DNA elements through mechanisms such as histone modification, DNA methylation and non-coding RNA regulation. Heterochromatin dysfunction can include abnormal repeat element repair, chromosome missegregation, transposon activation and replication stress, and is associated with some genetic disorders, aging, and tumor development (1).

Specific histone methylation or DNA methylation are important epigenetic features of heterochromatin domains. H3K9me3, H3K9me2 and H3K27me3 are significantly enriched in heterochromatin domains across most organisms and are considered markers of heterochromatin domains (2). As constitutive heterochromatin marks, H3K9me3 and H3K9me2 regulate the formation, intergenerational inheritance and stability of heterochromatin through processes such as deposition, recruitment of histone-modifying enzymes and aggregation of heterochromatin proteins (2,3). Moreover, H3K9 methylation plays a critical role in establishing and maintaining cell identity and tissue differentiation. Abnormal methylation of H3K9 leads to changes in chromatin structure and function, a feature of aging and certain diseases such as cancer (4,5). H3K27me3 is a core component of facultative heterochromatin that recruits Polycomb protein complexes to alter chromatin structure and tightly wrap chromatin in heterochromatin (6). Additionally, H3K27me3-mediated heterochromatin inhibits cell reprogramming and affects tissue development and cell fate through its self-reorganization (4,6). Abnormal modifications of H3K27me3 are associated with disease occurrence. For instance, H3K27me3 is significantly reduced in prostate cancer cells and is associated with invasive markers of prostate cancer (7). Furthermore, H3K27me3 levels can define subgroups of non-small cell lung cancer (NSCLC) patients with different epigenetic phenotypes and clinical outcomes, and can potentially serve as a novel prognostic marker for NSCLC patients (8). Heterochromatin-enriched regions are typically found in structural regions of the genome, such as telomeres and centromeres. However, genome-wide heterochromatin regions have become increasingly recognized. Studies have revealed significant differences and dynamics in heterochromatin-enriched regions among different cell types and tissues, emphasizing the importance of further exploration and discovery of additional heterochromatin regions (2).

To study heterochromatin functions, a few dedicated databases for heterochromatin have been developed, such as Genome Structure Database (GSDB) and 3D-genome Interaction Viewer and Database (3DIV). GSDB mainly relies on 3D modeling tools to predict chromatin structure, while 3DIV primarily stores chromatin interaction information. To overcome the shortcomings of existing resources, H3K9me3, H3K9me2 and H3K27me3 modifications, determined by the widespread application of chromatin immunoprecipitation sequencing (ChIP-seq) technology, can be used to accurately reflect chromatin activity and function.

We therefore developed the Human Heterochromatin Chromatin Database (HHCDB), which integrates H3K9me3, H3K9me2 and H3K27me3 modification data obtained through ChIP-seq technology. The version of HHCDB presented here assessed 42 839 743 heterochromatin regions identified in 241 cell types/cell lines and 92 tissues. HHCDB possesses several unique advantages for identifying heterochromatin regions: (i) we developed a unified pipeline for the identification of heterochromatin regions to avoid the noise caused by combining different methods; (ii) it provides abundant genomic information and annotation related to heterochromatin regions, including gene structure, transcripts, distance to transcription start sites (TSSs), neighboring genes, repetitive sequences, transposable elements, CpG islands and 3D genomics and functional annotations; (iii) heterochromatin regions are visualized using the UCSC genome browser and an in-house developed tool for transposable elements, CpG islands, 3D genomics, super enhancer and Dnase I; (iv) it can reflect gene expression associated with heterochromatin regions in different cell types of tissues using single-cell data and can display the level of gene expression by TSNE, UMAP and violin plots; (v) we created a heterochromatin region analysis platform that implements a differential heterochromatin identification algorithm. This integrated nine cancer transcriptome datasets from The Cancer Genome Atlas (TCGA) database. We also developed several tools to further explore the relationship between cancer-specific heterochromatin-related genes and clinical features, immune cell infiltration, drugs, transcription factors and protein-protein interactions. HHCDB provides an efficient data storage and mining platform with an interactive interface that can be used to study epigenetic regulation, support 3D genomics research, drug target discovery and the investigation of mechanisms that regulate heterochromatin in development and disease.

## HHCDB design

**Figure 1.**
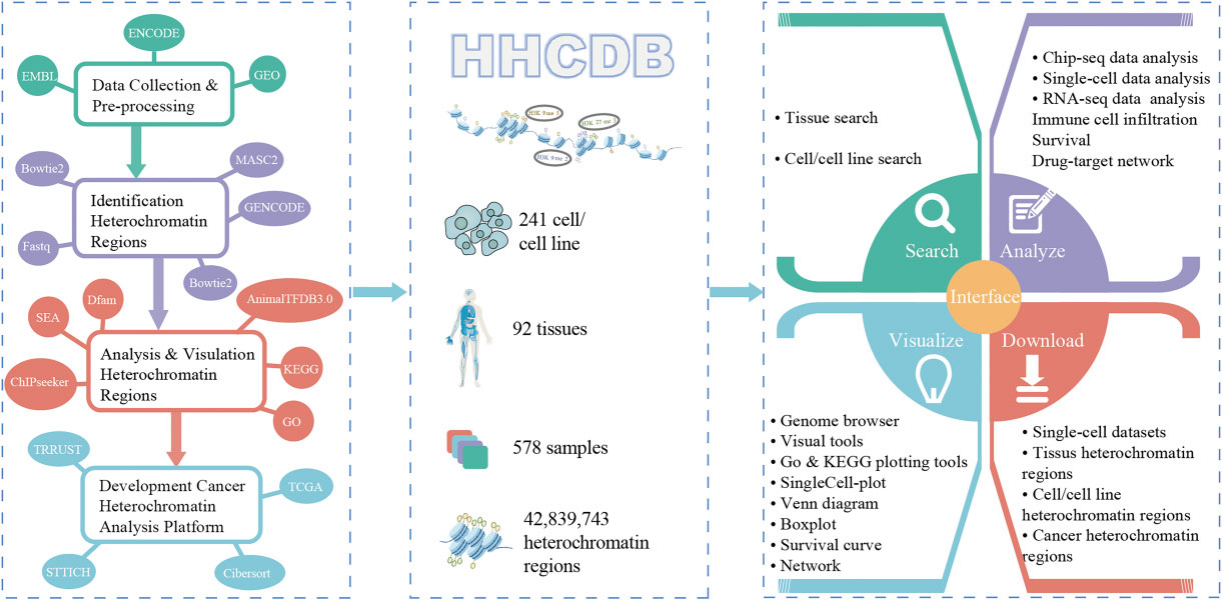
The overall framework of HHCDB.

We have developed a new human heterochromatin region database called HHCDB, which provides a unified pipeline for identifying heterochromatin regions by integrating H3K9me3, H3K9me2 and H3K27me3 histone modification data from publicly available datasets. It identified a total of 42 839 743 heterochromatin regions in 578 samples from 241 human cell types/cell lines and 92 tissue types. HHCDB also enables further exploration and in-depth analysis of heterochromatin regions of interest (Figure 1). Moreover, it provides a differential heterochromatin region analysis platform that can identify differential heterochromatin regions between case and control samples. Leveraging the storage and query methods of the MySQL database, users can swiftly access heterochromatin region information of any tissue or cell type/cell line. In addition, we developed a more extensive and user-friendly interface to provide a criteria query function, rich visualization, personalized analysis and data downloads, while ‘SingleCell-plot’ can display the expression of heterochromatin-related genes in different cells of a tissue. Data can be freely downloaded from the database website.

## Data collection and pre-processing

To construct a versatile database, we retrieved H3K9me3, H3K9me2 and H3K27me3 modification data captured through ChIP-seq from the Gene Expression Omnibus (GEO) (9), the Encyclopedia of DNA Elements Consortium (ENCODE) (10) and the European Molecular Biology Laboratory (EMBL) database (11) up to June 2023. Simultaneously, we obtained abundant genomic information related to human heterochromatin regions from different databases and tools. Gene structure, transcripts, TSSs and neighboring genes were annotated by R package ‘ChIPseeker’ (12). Repetitive sequences were implemented by the UCSC Genome browser (https://genome.ucsc.edu/) (13). Transcription factors and transposable elements were respectively collected from AnimalTFDB3.0 (http://bioinfo.life.hust.edu.cn/AnimalTFDB/) (14) and Dfam (https://www.dfam.org/home) databases (15). CpG islands and 3D genomic information were retrieved from the Super-Enhancer Archive version 3.0 (SEA v3.0) database (http://sea.edbc.org/) (16). Gene annotation files and hg38 reference genome data were downloaded from the GENCODE database (https://www.gencodegenes.org/) (17). Functional annotations were gathered from Gene Ontology (GO) (http://geneontology.org/) (18) and Kyoto Encyclopedia of Genes and Genomes (KEGG) (https://www.genome.jp/kegg/) databases (19).

To display the expression of heterochromatin-related genes in different cell types of a tissue, we searched for single-cell data from the GEO database using the keywords ‘tissue name scRNA-seq’ or ‘tissue name single cell’. In total, we collected 73 datasets, covering 349 samples and 90 tissues, each containing 65–12 170 genes. For single-cell datasets of each tissue, a standard process was applied to obtain cell annotations: low-quality cells were filtered out by RNA counts and mitochondrial gene percentage; data were normalized and integrated by R packages ‘Seurat’ (20) and ‘Harmony’ (21); clusters were annotated by R package ‘SingleR’ (22) and with markers obtained from the literature. In this way, the cell types of a tissue were annotated, and then the expression of heterochromatin-related genes was extracted. The single-cell datasets we analyzed can be downloaded from the download page.

Heterochromatin regions are crucial for understanding the molecular basis of many diseases, such as cancer. We used HHCDB to integrate transcriptome and clinical data from nine cancers in TCGA retrieved from UCSC Xena (https://xenabrowser.net/datapages/). Differentially expressed mRNAs [|log2FoldChange| ≥ 1, false discovery rate (FDR) < 0.05] were screened between cancer samples and normal samples using R package ‘limma’. We then analyzed the function of genes related to cancer-specific differential heterochromatin regions and their relationships with clinical features, immune cell infiltration, drugs and TF and protein interaction. The Kaplan-Meier method was applied to compare survival curves and P values were obtained using the log-rank test in the R package ‘survival’. The ‘Cibersort’ algorithm was employed to obtain the infiltration proportion of 22 immune cells (23). The relationships between drugs and targets were gained from STITCH (http://stitch.embl.de/) (24), the relationships between transcription factors and genes were gathered from TRRUST (https://www.grnpedia.org/trrust/) (25) and protein interaction information was extracted from String (https://cn.string-db.org/) (26). The sources of the data collected in our database are shown in Table 1.

**Table 1.** Statistics of the data in HHCDB

Data source	Data types	Sample number	Heterochromatin region (one histone modification)	Heterochromatin region (two histone modifications)	Heterochromatin region (three histone modifications)
GEO	11 (tissues)	20	483 001	\	\
	7 (cells)	12	458 149	\	\
	42 (cell lines)	57	986 091	\	\
	73 (single-cell data)	349	\	\	\
ENCODE	78 (tissues)	132	3 037 358	\	
	107 (cells)	185	26 714 405	\	\
	65 (cell lines)	135	9 676 827	\	\
EMBL	3 (tissues)	6	775 62	\	\
	4 (cells)	6	134 650	\	\
	16 (cell lines)	25	395 802	\	\
GEO &	64 (tissues)	\	\	64 589	\
ENCODE &	67 (cells)	\	\	395 643	1595
EMBL	87 (cell lines)	\	\	412 408	1663

## Standard pipeline and tools for the detection and analysis of heterochromatin regions

**Figure 2.**
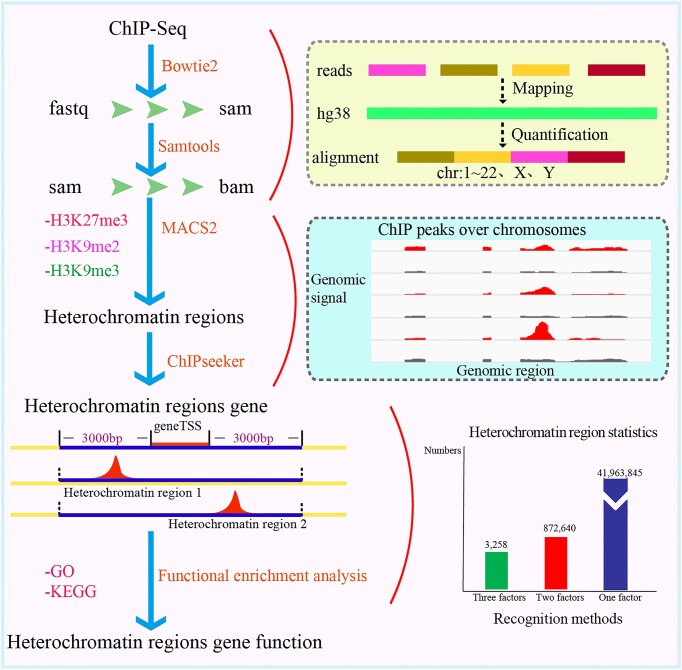
The pipeline to identify and annotate heterochromatin regions.

To facilitate the use of HHCDB and to avoid the noise caused by using a combination of different methods, a unified and standardized pipeline was developed for identification of heterochromatin regions. Using our pipeline, more than 42 839 743 heterochromatin regions were identified from H3K9me3, H3K9me2 and H3K27me3 histone modification data derived from 578 samples, 241 human cell types or cell lines and 92 tissue types. 41 963 845 of the heterochromatin regions were recognized by single histone modification (H3K9me3, H3K9me2 or H3K27me3), 872 640 heterochromatin regions were recognized by a combination of two histone modifications (H3K9me3 & H3K9me2, H3K9me3 & H3K27me3 or H3K9me2 & H3K27me3), and 3258 heterochromatin regions were recognized by a combination of three histone modifications (H3K9me3 & H3K9me2 & H3K27me3). The pipeline consists of the following steps (Figure 2):

- Download raw data, narrowPeak format data, hg38 reference genome data, gene annotation files, transcription factor target information, transposable elements, CpG islands, 3D genomics, GO and KEGG pathway data from public databases.
- Perform quality control for the raw data using fastq (27).
- Construct a reference genome index and perform alignment using Bowtie2 (http://bowtie-bio.sourceforge.net/bowtie2) (28).
- Convert sam files to bam files and build index files using samtools (29).
- Detect heterochromatin region peaks using MACS2 (30) and classify heterochromatin regions. The heterochromatin regions can be classified according to three situations: (a) recognition by one of three histone modifications (H3K9me3, H3K9me2, or H3K27me3). (b) Recognition by two histone modifications (H3K9me3 & H3K9me2, H3K9me3 & H3K27me3, or H3K9me2 & H3K27me3); when the overlapping region of two histone modifications is greater than 50% of any one region, this region is considered to be recognized by the two histone modifications. (c) Recognition by three histone modifications (H3K9me3 & H3K9me2 & H3K27me3); if the overlap among three regions is greater than 50% of the length of any one region, then this region is considered to be jointly recognized by three histone modifications.
- Perform annotation of heterochromatin regions using the R package ‘ChIPseeker’, with the tssRegion parameter set to [-3000, 3000] (12). ‘ChIPseeker’ is an R package for annotating ChIP-seq data. It implements functions to retrieve the nearest genes around the peak and to annotate the genomic region of the peak. We therefore used ‘ChIPseeker’ to annotate heterochromatin regions, and the annotated genes were considered to be associated with heterochromatin regions.
- Convert the data for heterochromatin regions into gene symbols and annotated transcription factors, GO IDs and KEGG IDs.

**Figure 3.**
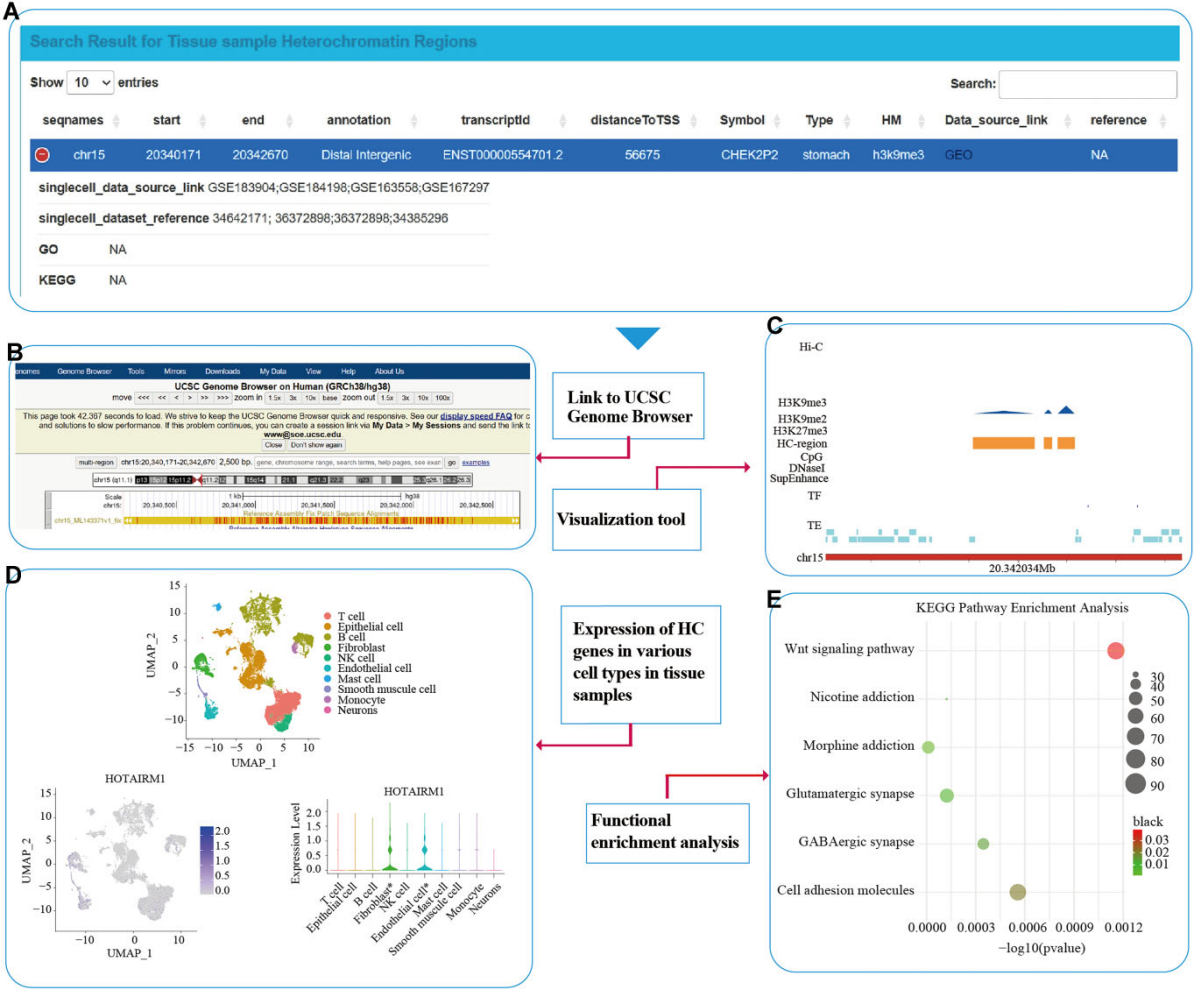
A case application of the HHCDB search function. **(A)** Table obtained after a query. **(B)** Display of heterochromatin (HC) regions in the UCSC genome browser. **(C)** Our in-house heterochromatin region visualization tool. **(D)** Heterochromatin gene expression in various cells from tissue samples. **(E)** Functional enrichment analysis.

We have applied and developed many tools to further explore heterochromatin regions obtained through the aforementioned HHCDB pipeline (Figure 3). The UCSC genome browser was applied to visualize heterochromatin regions. Furthermore, we also developed a visualization tool that can display transposable elements, CpG islands, 3D genomics and peaks associated with heterochromatin regions. The raw histone modification peaks by ChIp-seq for a given heterochromatin region were displayed in the visualization views. In our visualization tool, it can also be clearly seen that the heterochromatin region is recognized by several histone modifications. At the same time, we will also display active components such as super enhancers and DNase I in our visualization tools. GO and KEGG functional enrichment analysis were also implemented for genes associated with the obtained heterochromatin region, allowing users to flexibly select the corresponding thresholds. For tissue samples, an additional analysis tool called ‘SingleCell-plot’ was created. For the collected single-cell data, a standard single-cell workflow was applied and the gene expression associated with heterochromatin regions was extracted. For each heterochromatin-associated gene expressed in the corresponding single-cell dataset, TSNE, UMAP and violin plots were generated to reflect their expression in different cell types of the tissue.

## HHCDB search section

In the search interface, there are two search approaches available, ‘Search by Tissues’ and ‘Search by Cells OR Cell lines.’ In the input box of ‘Search by Tissues,’ recognition factors and corresponding tissue names are required, and ‘Gene,’ ‘Chromosome,’ ‘Starting site,’ ‘Termination site,’ ‘GO ID’ and ‘KEGG ID’ are optional. Inputting one of the eight options for recognition factors (‘H3K9me3’, ‘H3K9me2’, ‘H3K27me3’, ‘H3K9me3 & H3K9me2’, ‘H3K9me3 & H3K27me3’, ‘H3K9me2 & H3K27me3’, ‘H3K9me3 & H3K9me2 & H3K27me3’ and ‘ALL’) and corresponding tissue names, genomic information, including gene structure, transcripts, TSS, neighboring genes, GO ID and KEGG ID associated with a heterochromatin region can be displayed in an intuitive tabular format. The corresponding references and source for each heterochromatin region are also displayed in the table. Additionally, we also linked the UCSC genome browser to enable visualization of heterochromatin regions obtained by querying. Transposable elements, CpG islands, 3D genomics, super enhancers, Dnase I and peaks associated with heterochromatin regions can be displayed by our visualization tools. The results of GO and KEGG functional enrichment analysis for heterochromatin-related genes obtained by querying are also enabled. At the same time, in the query interface, users can freely select the threshold to obtain different GO and KEGG functional enrichment results. On the ‘Search by Cells OR Cell lines’ page, the query method and the display format of the returned results are the same as for ‘Search by Tissues.’ Several of the special tools described above are also suitable for ‘Search by Cells OR Cell lines.’

For ‘Search by Tissues,’ ‘SingleCell-plot’ is available. Clicking on the ‘Show’ button of ‘SingleCell-plot,’ shows TSNE and UMAP plots of cell annotations for the queried tissues, as well as the TSNE, UMAP and violin plots of the queried gene. They are displayed to represent the cell composition of the tissue and reflect the abundance of gene expression in different cell types of the tissue. It is worth noting that asterisks (*) will appear in the violin plots if a gene is a cell marker. There are no corresponding violin, UMAP and TSNE plots for queried genes that are not expressed in the single cell dataset. All data and generated images can be downloaded from the search interface and the heterochromatin regions of tissues or cell types/cell lines and the single-cell datasets we used can be downloaded on the download interface.

## Cancer heterochromatin analysis platform development

Heterochromatin is a key driver and potential biomarker of cancers. Therefore, we developed a differential heterochromatin identification algorithm to identify differential heterochromatin regions, termed Differential Heterochromatin Region Analysis Platform (DFRAP). The algorithm formula is:


\begin{equation*}F\left( {Regio{n}_{Tj}} \right) = \left\{ {\begin{array}{@{}*{1}{c}@{}} {\ 1,Regio{n}_{Tj} \cap \ Regio{n}_C = \emptyset }\\ {M,Regio{n}_{Tj} \cap \ Regio{n}_C \ne \emptyset } \end{array}} \right.\end{equation*}

\begin{equation*}M = \left\{ {\begin{array}{@{}*{1}{c}@{}} {1,\ Pvalu{e}_j \le 0.05}\\ {0,Pvalu{e}_j >0.05} \end{array}} \right.\end{equation*}

$j = 1,2, \ldots ,N$ : represents the index of N heterochromatin regions in the dataset. $Regio{n}_T$: denotes the heterochromatin region of case samples. $Regio{n}_C$: signifies the heterochromatin region of control samples. $Pvalue$: the *P*-value is derived from the R package ‘edgeR’, which compares peak differences in regions where case and control samples coincide. $F( {Regio{n}_{Tj}} )$: a binary function used for the classification of each $Regio{n}_{Tj}$. Specifically, when $F( {Regio{n}_{Tj}} )$ equals 1, $Regio{n}_{Tj}$ is identified as a significantly different heterochromatin region in the context of the case sample. Conversely, when $F( {Regio{n}_{Tj}} )$ equals 0, $Regio{n}_{Tj}$ manifests that the heterochromatin region was not associated with the case sample. In summary, the F function classified each $Regio{n}_{Tj}$, distinguishing heterochromatin regions that displayed significant differences associated with the case sample ($F( {Regio{n}_{Tj}} ) = 1$) from those that did not ($F( {Regio{n}_{Tj}} ) = 0$). The algorithm aided in the identification of specific heterochromatin regions that are potentially implicated in the case sample.

To identify crucial targets in cancers, we combined 271 cancer-specific heterochromatin regions obtained by this algorithm from nine cancers with corresponding transcriptome data from the TCGA database. Cancer-specific heterochromatin genes were identified by intersecting cancer differentially expressed genes with genes related to cancer-specific heterochromatin regions.

**Figure 4.**
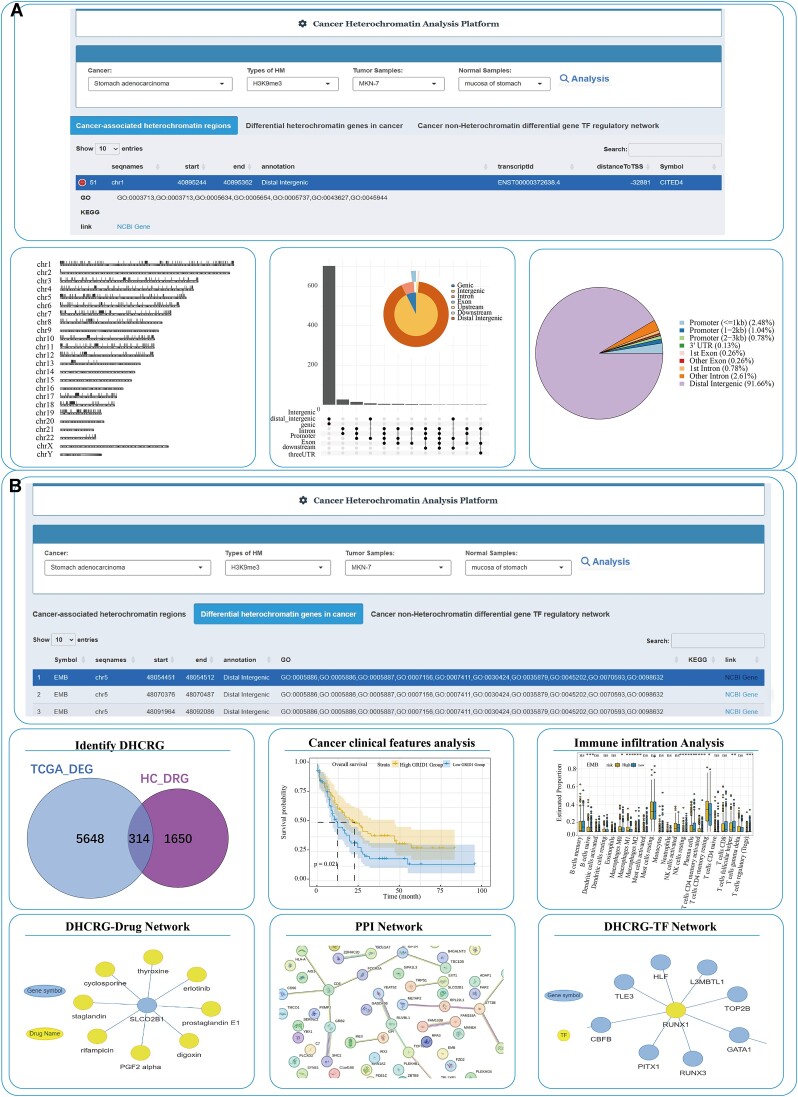
Overview of the Cancer Heterochromatin Analysis Platform in HHCDB. **(A)** Display of differential heterochromatin regions (DHCRs) in cancer and visualization of their genome-wide distribution. **(B)** Identification, display and online analysis of differential heterochromatin region genes (DHCRGs) in cancer, including clinical features analysis, immune infiltration analysis, TF-gene network, drug-gene network and protein–protein interaction network.

A user-friendly analysis interface is provided to help users identify cancer-specific heterochromatin regions and to explore the importance of cancer-specific heterochromatin genes. Users have the flexibility to customize the input box by selecting cancer type, identifying factor, cancer sample and normal sample. Similar to the result of tissue and cell type/cell line queries in the search interface, corresponding genomic information, including gene structure, transcripts, TSS, neighboring genes, GO ID and KEGG ID associated with cancer-specific heterochromatin regions can be displayed in an intuitive tabular format. The genomic distribution of cancer-specific heterochromatin regions can also be displayed. In the analysis interface of cancer-specific heterochromatin genes, cancer-specific heterochromatin genes appear in a table and Venn diagrams are provided to display the intersection between differentially expressed genes in cancer and genes in cancer-specific heterochromatin regions. Furthermore, we provide various analysis tools to explore the functions of cancer-specific heterochromatin genes, including association with clinical features and immune cell infiltration, construction of drug–target networks and protein–protein interaction networks. For cancer non-heterochromatin genes, a transcription factor regulatory network can be constructed. All analysis data and generated images are available for download (Figure 4).

## System design and implementation

HHCDB was built upon three main software components: the shiny-server, the MySQL (https://www.mysql.com/cn/) relational database and R-based computing services. The backend processing programs and frontend interface were both developed using the R package, shiny.

## Discussion and future development

The eukaryotic genome is partitioned into euchromatin and heterochromatin, with most studies focused on the former (31). However, heterochromatin has essential roles in nuclear architecture, DNA repair and genome stability, transposon silencing and gene expression (32). In-depth knowledge of molecular and genetic features of heterochromatin can reveal complex interactions between chromatin structure, gene expression and cellular processes, thereby promoting the understanding of developmental biology and disease (2). Heterochromatin was originally identified by differential staining with DNA dyes. Nowadays, heterochromatin can be defined by multiple chromatin modifications, such as H3K9me2, H3K9me3 and H3K27me3 (2,31). With the widespread accumulation of ChIP-seq sequencing data, there is a wealth of human H3K9me2, H3K9me3 and H3K27me3 histone modification data in public databases. Therefore, the development of a human heterochromatin region database based on histone modification marks is necessary and feasible. Here, we have developed HHCDB, which integrates human H3K9me3, H3K9me2, and H3K27me3 modification data obtained through ChIP-seq technology to identify heterochromatin regions. The version of HHCDB presented here assessed 42 839 743 heterochromatin regions identified in 241 cell types/cell lines and 92 tissues.

HHCDB is currently the only specialized human heterochromatin database. We developed a unified pipeline for HHCDB to identify heterochromatin regions to avoid the noise caused by combining different methods. Heterochromatin is enriched for repetitive elements and epigenetic marks, which affect genome stability and result in transposon and gene expression silencing. This genomic information, including gene structure, transcripts, TSS, neighboring genes, repetitive sequences, transposable elements, CpG islands and 3D genomics are also provided in HHCDB. In addition, annotated information and rich visualizations are also displayed. Of particular note is the integration and analysis of 73 single-cell transcriptome datasets, which enables users to explore cell type-specific heterochromatin-associated genes and their functions, thereby enhancing our understanding of genes associated with heterochromatin regions.

Heterochromatin plays a crucial role in tumors, influencing gene expression, genome stability, epigenetic regulation and tumor response to treatment. A comprehensive understanding of heterochromatin is important for unraveling mechanisms of tumor progression, developing novel therapeutic strategies and advancing personalized cancer treatments. Therefore, we developed a differential heterochromatin region analysis platform in HHCDB. In addition to the implementation of a differential heterochromatin identification algorithm, it supports the analysis of cancer-specific heterochromatin-related genes in terms of their function and their association with clinical features, immune cell infiltration and drugs. HHCDB offers users a fresh perspective on the role of heterochromatin in cancer, and provides a powerful tool to identify cancer-specific heterochromatin biomarkers that may help the diagnosis and treatment of cancer. It is worth noting that the differential heterochromatin algorithm we developed is not limited to the identification of cancer-specific heterochromatin regions; it can also identify any disease-specific heterochromatin region of interest. In conclusion, HHCDB is a valuable resource for studying epigenetic regulation, 3D genome functionality, discovering drug targets, and investigating the regulatory mechanisms of heterochromatin in development and disease.

In the future, with the development of single-cell histone modification detection technology, such as Paired-Tag (33,34) and single‐cell ChIP-seq, more and more data will be generated and the understanding of heterochromatin will become more profound. We will continue our efforts to ensure that HHCDB remains up-to-date. Through continuous use and improvement of HHCDB, we hope to deepen our understanding of the role heterochromatin plays in disease, cell differentiation, and tissue development.

## Data Availability

The HHCDB database is publicly accessible at (http://hhcdb.edbc.org/). Within HHCDB, all charts and tables are available for free download. Users have unrestricted access to all data in HHCDB.
